# The Processing of Color Words in Sentence Comprehension

**DOI:** 10.1177/17470218261428130

**Published:** 2026-02-20

**Authors:** Emily L. Buchner, Tobias Richter, Wolfgang Lenhard

**Affiliations:** 1 Department of Psychology IV, University of Würzburg, Germany

**Keywords:** color representation, perceptual simulations, reading, sentence comprehension

## Abstract

This study investigated how color is represented in language comprehension. Some theories suggest that perceptual simulations—activations of sensory features such as shape, size, or color—routinely support understanding during reading. One line of evidence is the “mismatch effect” in sentence-picture verification tasks: responses are slower when pictures mismatch perceptual details described in the sentence. Across three preregistered experiments (*N* = 222), we tested whether this mismatch effect occurs with explicit color words (Experiment 1) and whether background colors presented concurrently interfere with the mental simulation of color to test the functionality of mental simulations in language comprehension (Experiments 2 and 3). As expected, participants responded faster when pictures matched the sentence’s color across the three experiments. When conflicting background colors were introduced during sentence presentation, the mismatch effect remained unaffected. This pattern of findings suggests that colors are routinely activated through color words during comprehension, but the functional role of perceptual simulations of color for comprehension remains unclear.

## Introduction

Consider the following example: “Timothy opens the blue lid of the lunchbox and sees a green layer where he had placed a clementine a fortnight ago.” The sentence contains both colors that are explicitly named (blue, green) and a color that is implicitly associated (clementine, typically orange). Understanding these color references enables readers to represent what the lunchbox looks like and to infer that the clementine has grown moldy.

Some theories of language comprehension assume that linguistic information activates multimodal networks that store experiential properties, resulting in a multimodal simulation (Barsalou, 1999, 2008; Zwaan, 2004). Applied to the comprehension of color information, a color word should lead to a simulation of the described color. One way to examine the assumption that perceptual representations are activated during language comprehension is the sentence-picture verification paradigm (Stanfield & Zwaan, 2001). In this paradigm, pictures are presented after sentences, and the participants’ task is to judge whether the object depicted in the picture appeared in the sentence. By varying perceptual properties such as the orientation (Stanfield & Zwaan, 2001) or the color of the picture (Hoeben Mannaert et al., 2017) studies have shown that the verification response is faster for matching than for mismatching perceptual properties, which has been interpreted as meaning that perceptual properties are routinely activated during sentence comprehension (in line with Barsalou, 1999). Furthermore, neuroimaging studies have demonstrated that the brain areas recruited for processing perceptual properties overlap with brain areas recruited for processing words referring to these properties (Kosslyn et al., 2001; for colors: Simmons et al., 2007; Van Dam et al., 2012). Nevertheless, it remains unclear whether perceptual representations are functional for comprehension or merely a by-product of the core comprehension process.

The present experiments examined the role of perceptual simulations of color in sentence comprehension with the two primary objectives of investigating whether color information is routinely activated when explicit color words are provided and investigating the functional role of perceptual simulations in the comprehension of color words. In the following sections, we briefly review the current state of the literature and previous methodological approaches to investigating color representations in language comprehension. Based on this review, we derived specific research questions, which we addressed in three experiments employing sentence-picture verification tasks. First, we examined whether the well-established mismatch effect also applies to color words (see Hoeben Mannaert et al., 2017, 2021; Zwaan & Pecher, 2012, for implicit color information). Second, we introduced conflicting visual color information concurrently with sentence processing to gain insights into the mechanisms underlying mental simulations. Comprehension effects were assessed in terms of response latencies and accuracy.

### Studies Using the Sentence-Picture Verification Paradigm to Study the Role of Color in Comprehension

According to the theory of perceptual symbol systems (Barsalou, 1999, 2008), perceptual properties of an object are routinely represented mentally when language is comprehended. An established method for investigating the representation format of language is the sentence-picture verification task (cf. Stanfield & Zwaan, 2001): Participants read a sentence including an object that implies certain perceptual properties. They then see a picture that either matches or mismatches the perceptual properties of the object. Their task is to judge whether the depicted object was mentioned in the sentence. Typically, this results in the so-called “match advantage” (Hoeben Mannaert et al., 2017), also known as the mismatch effect (Zwaan et al., 2002), which means that responses to the pictures that represent incongruent perceptual properties are slower than to the pictures that represent congruent perceptual properties. The effect is interpreted as indicating a routine activation of perceptual properties and as support for the assumption that comprehension processes are accompanied by perceptual simulations. The mismatch effect has been demonstrated across multiple perceptual dimensions (for a collection of experiments, see Zwaan & Pecher, 2012) such as object shape (de Koning et al., 2017a; Engelen et al., 2011; Zwaan et al., 2002) or size implied in sentences (de Koning et al., 2017a, 2017b; Koster et al., 2019).

For the perceptual dimension of color, the results have been less conclusive. Color is considered a perceptual dimension defined unambiguously by visual properties. That is, its representation is assumed to not be compensated by nonvisual modalities (Ostarek & Bottini, 2021). The investigation of this perceptual dimension could therefore be of particular interest for uncovering simulation mechanisms. In the past, however, inconsistent results were obtained when using sentence-picture verification tasks with objects that imply certain colors (color-diagnostic objects such as coal, tomatoes, or leaves). Contrary to the mismatch effect found for other perceptual dimensions, Connell (2007) found faster responses to incongruent combinations compared to congruent combinations. However, in a direct replication of Connell’s study (using the same stimulus material) Zwaan and Pecher (2012) found a mismatch effect, which is consistent with results usually obtained for other perceptual dimensions. As one possible explanation for these divergent results, Liu et al. (2024) discussed whether individual differences exist in the representation of implicit color information (e.g. green vs. orange for a leaf) because of differences in prior experiences. Another possibility is that color is processed and represented in a different way, as some studies showed that color is represented less stable or requiring more attentional resources than the other perceptual dimensions during comprehension (for a discussion, see Connell, 2007). In a conceptual replication based on a new stimulus set (again with implicit color information), the mismatch effect for color was replicated again (Hoeben Mannaert et al., 2017, Experiment 1). Along the same lines, a comparison of different perceptual dimensions (specifically shape, size, color, orientation) also yielded a greater mismatch effect for color compared to the other dimensions investigated (de Koning et al., 2017a). Furthermore, color representations were found to be rich in perceptual detail as the mismatch effect disappeared when the saturation of the picture color was reduced (Hoeben Mannaert et al., 2017, Experiments 2a and 2b).

To summarize, the majority of studies support the perspective that perceptual simulations of colors are routinely activated—at least for implicit color information. However, individual differences in people’s past experiences might be particularly relevant for colors, which could explain the contradictory results (cf. Connell, 2007; Zwaan & Pecher, 2012). Additional insights into the processing of verbal color information could be gained by investigating explicit color words for which individual differences in past experiences should be less relevant. To our knowledge, the only study using explicit color words in sentence-picture verification tasks was conducted by Hoeben Mannaert et al. (2021) who investigated the activation of color across multiple sentences. They varied sentences with explicit versus no color references and with pictures that showed a color versus a gray-scale variant of the focal object. Instead of employing the classical mismatch effect, they investigated a color advantage, that is, whether participants responded faster to colored pictures when a color reference was included in the sentence than to pictures with greyscale, whereas no difference in response latencies was expected when the sentence contained no color reference. The color advantage appeared as expected, even across multiple sentences but only when the focus was on the target object.

### Overlaps in the Visual Processing of Colors and the Comprehension of Color Words

According to Barsalou’s (1999, 2008) theory, the activated brain areas for the visual processing and the word-based processing of perceptual properties should overlap. In support of this assumption, activations in color processing areas were found for the reception of explicit color words (Simmons et al., 2007) and for implicitly associated color properties (Van Dam et al., 2012), which can be clearly distinguished from activations elicited by other perceptual dimensions (for a comparison with form words, del Prado Martín et al., 2006; Pulvermüller & Hauk, 2006). Studies have shown that words activate brain areas associated with their perceptual word meaning for other dimensions. For example, when the meaning of words is associated with visual properties, the visual cortex is activated during the processing of the words (Kosslyn et al., 2001; Mak & Willems, 2021; Pulvermüller, 1999). Behavioral experiments extended these correlational findings by demonstrating that the presentation of competing visual stimuli can interfere with the recall of visual knowledge associated with words (Edmiston & Lupyan, 2017) and impair the speed and accuracy of semantic judgments on words for which the content is associated with visual characteristics, with visual interference being more effective the more subjective visual experience the participants had with the object (Davis et al., 2020). This methodological approach has also been applied to studying the functionality of perceptual simulations in language comprehension, most explicitly for the perceptual dimension of shape (Ostarek et al., 2019) and dynamic directions (Buchner et al., 2025), but to our knowledge not yet for the dimension of color.

However, behavioral studies based on other paradigms that leverage interference also suggest an interplay between the processing of colors and color words. One example is the Stroop effect (Stroop, 1935; for an overview, see MacLeod, 1991), which is characterized by an increase in errors and processing time when naming the ink color of a presented word that is incongruent with the color word. However, this design addresses attentional processes, the automaticity of reading processes compared to color naming, and the parallel processing of relevant and irrelevant stimuli (for a discussion, see MacLeod, 1991) rather than the mental representation elicited by the color words. In the context of sentence comprehension, a modified semantic Stroop task was used by Connell and Lynott (2009) to investigate which colors are mentally represented when a sentence only implies color information (e.g. a bear at the North Pole indicating a bear with white fur). After participants read a sentence, the object word appeared again written in colored ink. The naming of the ink color was slower when the color mismatched the information from the previous sentence compared to when naming matching colors. Thus, the processing of implied color information appeared to facilitate color processing of visually congruent colors. A variety of Stroop-associated interference tasks have been developed, some of which have been extended with priming paradigms and expanded to more general picture-word interference (for an overview, see Glaser, 1992). Word-color interference tasks in which color primes are followed by color words to form congruent and incongruent trials (Cui et al., 2007; replication study by Azañón et al., 2025) are particularly informative for the present experiments. These studies demonstrated that trials with congruent color primes and color words lead to faster and more accurate responses compared to incongruent trials (see Azañón et al., 2025). Furthermore, Berndt et al. (2020) found that color-congruent primes (e.g. green) reduced the solution time for anagrams (e.g. *cbemcuru* for *cucumber*), which the authors interpreted as evidence for an important role of multimodal experiential traces in language comprehension.

These results are extended by the findings of Richter and Zwaan (2009) who found bidirectional congruency effects in the processing of visually perceived colors and color words. They combined a color discrimination task with a presentation of matching or mismatching color words on which lexical decisions were made. Lexical decisions on color words were found to be slower when the color word was incongruent with the color seen before compared to congruent combinations. In addition, color discrimination judgments were found to be slower when the colors were incongruent with a color word seen before compared to congruent combinations. This effect persisted when the lexical decision task was omitted, suggesting that color representations are activated routinely when color words are read.

To summarize, neuro-imaging and behavioral evidence suggests that the simulation of color properties based on words and the representation of colors based on perceptual impressions are interconnected. However, the question of the extent to which perceptual simulations are functional for comprehension remains unclear. Existing literature lacks experimental approaches that inhibit the simulation of color and objectify the effects on comprehension measures.

### Rationale of the Present Experiments

The present experiments were designed to examine the representation of color in sentence comprehension. We pursued two objectives. The first objective was to investigate whether the mismatch effect, that is, incongruent perceptual information in a sentence–picture verification task leads to impaired object recognition compared to congruent perceptual information (cf. Zwaan & Pecher, 2012), occurs for the perceptual dimension of color when sentences contain explicit color words (Experiment 1). We expected that responses in a sentence–picture verification task would be significantly slower (Hypothesis 1) and less accurate (Hypothesis 2) when the color of the object shown (picture color) and the color explicitly mentioned in the preceding sentence (color word) were incongruent compared to when the picture color and the color word were congruent.

The mismatch effect may be interpreted as evidence for the activation of modal representations in comprehension (Stanfield & Zwaan, 2001; Zwaan et al., 2002; Zwaan & Pecher, 2012) and as an indicator for mental simulations including multiple perceptual object properties (Hoeben Mannaert et al., 2017), but it cannot be interpreted as evidence for a functional role of mental simulations for successful comprehension (Mahon & Caramazza, 2008; for an overview, see Ostarek & Huettig, 2019). Therefore, the second objective was to gain a better understanding of the functionality of perceptual simulations in sentence comprehension. Neuroimaging studies indicate overlapping brain activation for language comprehension and perceptual processing (Kosslyn et al., 2001; Mak & Willems, 2021; Pulvermüller, 1999). This observation has also been made for color words and color processing areas (Simmons et al., 2007) as well as for words with specific color properties and color processing areas (Van Dam et al., 2012). These findings are correlational, but some experimental evidence also shows that the retrieval of visual information associated with the word meaning can be disrupted by visual stimuli (for single words, see Davis et al., 2020; Edmiston & Lupyan, 2017; Ostarek & Huettig, 2017; Richter & Zwaan, 2009). For color representations, these findings lead to the assumption that the presentation of visual color information can disrupt the simulation of word-based color information because both sources of information compete for the same cognitive resources.

In Experiment 2, we used the same sentence-picture verification task from Experiment 1 but also included an experimental manipulation of color during comprehension. Colored background screens during the sentence presentation (background color) were used to visually display a congruent or incongruent color relative to the color word. If perceptual simulations are indeed functional for comprehension, an incongruent background color should hamper comprehension of the color words, leading to slower and less accurate responses in the sentence–picture verification task compared to a congruent background color. We expected slower and less accurate responses in the verification task when the background color was incongruent with both the color word and the picture color (incongruent background color condition, Hypotheses 1a and 1b) and when the background color was incongruent with the color word but congruent with the picture color (incongruent color word condition, Hypotheses 2a and 2b). In the latter condition, the background color is likely to prime the congruent picture color, which should facilitate “yes” responses in the verification task, potentially reducing the interference introduced through the incongruent color word. Therefore, we expected responses in the incongruent background color condition to be slower and less accurate than in the incongruent color word condition (Hypotheses 3a and 3b). Experiment 3 was conducted to replicate the findings from Experiment 2. All experiments were preregistered.

The preregistered hypotheses focus on off-line measures of reading comprehension. However, given that mental simulation is assumed to develop during the reading process (Zwaan, 2004; Zwaan et al., 1998), interference from visual background color may exert its influence during on-line processing. Such interference could vary depending on how deeply participants engage with the sentence. Therefore, considering reading time may offer additional insights into the on-line simulation processes and the effect of visual manipulation on the expected mismatch effect. As an exploratory research question in all experiments, we examined the extent that reading time—as an indirect index of processing effort during sentence reading—predicts response accuracy and/or response latency in a condition-specific manner in the sentence-picture verification task.

## Experiment 1

Experiment 1 used a sentence-picture verification task to investigate the representation of colors in sentences with explicit color words (for the preregistration, see https://aspredicted.org/w6qk-4rtq.pdf). We expected that when the picture color and the color word are incongruent, participants would respond slower (Hypothesis 1) and less accurately (Hypothesis 2) than when the picture color and the color word are congruent.

### Method

#### Participants

We conducted an a-priori power analysis for a one-tailed *t*-test as an approximation of the planned (Generalized) linear mixed models (LLM), under the expectation of a small effect of Cohen’s *d* = 0.30 (cf. Zwaan et al., 2002), a Type I error probability of α = .05, and a power of 1–β = .80. Adding a margin of 20% for prespecified exclusions and to acknowledge that (Generalized) LLMs may require a larger sample, this resulted in a required sample size of *N* = 84 (according to G*Power, Faul et al., 2007). The following preregistered exclusion criteria were applied in all three experiments: First, participants were only eligible to take part in the experiment when they were able to speak and understand German on a native level and were able to fully distinguish colors. Second, participant data were excluded when less than 80% (cf. Hoeben Mannaert et al., 2017, 2021) of the sentence-picture verification task responses were correct. Third, we preregistered that when the average accuracy for individual sentence–picture combinations was below 80%, this combination was excluded for analyses (cf. Hoeben Mannaert et al., 2017). Fourth, we log-transformed response latency (ms) and only included data within a range of *M* ± 2 standard deviation (*SD*) of the log-transformed response latency based on group means. The third criterion did not apply to any of the three experiments reported in the current study.

Four participants were excluded because of the second exclusion criterion. Eighty participants were included in the analysis (age: *M* = 25.33 years, *SD* = 8.70; 65 female, 14 male, 1 diverse). The majority of the participants were students (*n* = 73) of which 20 were psychology students, 34 were teacher students, and the remainder (*n* = 19) were enrolled in other study programs. The participants were offered course credit or monetary reward for participation in all experiments.

#### Materials

##### Sentences

We created 72 German sentences for the sentence–picture verification tasks (following Stanfield & Zwaan, 2001). Each sentence mentioned a focal object. None of the objects implied a specific color but were color-neutral (e.g. a ball, a jumper, an umbrella) and were complemented by a color word. The color words appeared either in the third or sixth position of the sentences, restricted by the grammar of German. We used four color words (yellow, red, blue, green), distributed equally across the two sentence structures. Each sentence consisted of seven words.

##### Pictures

Pictures corresponding to the sentences were created with the illustration software SketchBook (Sketchbook, Inc., version 5.1.0). The pictures depicted objects schematically but in color. The colors used in the procedure represented pure instances of the Red, Green, Blue (RGB) color model for yellow (255, 255, 0), red (255, 0, 0), green (0, 255, 0), and blue (0, 0, 255), which were related to the four colors described in the sentences.

For experimental trials, the pictures represented the objects described in the corresponding sentences. Each picture existed in two color versions: congruent to the color word in the corresponding sentence and incongruent to the color word in the corresponding sentence. For filler trials, the pictures represented objects other than those described in the corresponding sentences. The colors illustrated in the filler pictures were always congruent with the color word in the corresponding sentence (to prevent strategic responding, cf. van Zuijlen et al., 2024).

##### Pilot Study

The stimuli were pilot-tested with an independent sample of 10 participants. The aim of the pilot study was to validate whether the objects visualized in the pictures were identifiable in combination with the experimental sentences. One participant responded “not at all” to the question “Was it clear to you what the pictures were supposed to represent?” and was thus excluded. Nine participants were included (age: *M* = 22.44 years, *SD* = 2.96; 8 female, 1 male). We presented 84 sentence-picture combinations, divided into 56 experimental trials and 28 filler trials. Participants read a sentence followed by a picture that depicted the congruent color. They were asked to judge whether the object visualized matched the object described in the preceding sentence. Participants were instructed to press “j” when the object matched the object described in the sentence and “f” when it did not match. When less than 85% of the participants gave an unambiguous answer for the experimental sentence–picture combinations, this combination was revised for the main experiments. This procedure resulted in the revision of five sentence-picture combinations. One additional object (“eyeshadow”) was revised because we received feedback from participants that this word could cause gender-specific differences in response behavior.

We also asked for a general assessment of the sentences and pictures, using a 7-point Likert scale (0 = *not at all*; 7 = *very good*). The quality of the pictures (*M* = 6.56, *SD* = 0.72) and the comprehensibility of the sentences (*M* = 6.11, *SD* = 0.93) were rated as high. We also asked whether the number of sentences and pictures was acceptable. Agreement was very high (*M* = 6.44, *SD* = 1.33).

##### Dependent Variables

Dependent variables were response latency and response accuracy in the sentence–picture verification tasks. Analyses of response latency were based on log-transformed response times (in ms), and only correct responses were included.

#### Procedure

The experiment was conducted in the laboratory with the software Inquisit Lab (version 6.6.1, Millisecond, 2021). Participants sat in front of a computer with an external screen and a keyboard. Participants received the instructions in written form via the computer screen, gave their informed consent, and provided demographic data. We conducted a brief color diagnostic, which was explained to the participant as a keyboard test because we tried not to draw attention to color reception throughout the communication of the experiment to avoid unintended effects. In this context, two pictures from the ensemble of the Ishihara test (for a discussion and evaluation, see Birch, 1997) and two pictures showing letters appeared. Participants were required to press the corresponding keys for the numbers and letters on the screen, which ensured that all participants were able to discriminate colors beyond self-assessment.

The instructions for the experimental task followed Hoeben Mannaert et al. (2021). Participants should rate whether the object mentioned in the sentence was depicted in the picture. However, they were instructed to disregard the color in their judgments. Responses were to be given via key press (“j” for “true” or “f” for “false”) with the index fingers of the left and the right hands. Furthermore, participants were instructed that each word was presented separately and that they should press the space bar with their thumb when they wanted to proceed to the next word. As in Zwaan et al. (2002), participants were told that they should respond as quickly as possible because their response times would be measured. After reading the written instructions, participants practiced the task on eight sentence–picture combinations with feedback (as in Hoeben Mannaert et al., 2017; Stanfield & Zwaan, 2001) and afterward started with the main experiment. We reminded the participants to place their fingers on the relevant keys of the keyboard.

**Figure 1.**
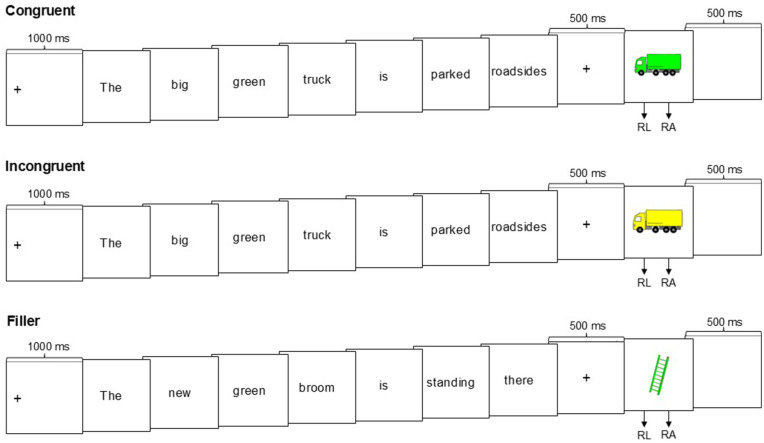
Example of the trial sequence in Experiment 1. *Note.* The same experimental sentence was combined with a congruent picture (“Congruent”) and an incongruent picture (“Incongruent”) but only appeared once per list and per participant. The sentences were presented in German. The English translations of the sentences may not be exact, as they were intended to contain seven words like the German originals (original versions: “Der große grüne LKW steht am Straßenrand” and “Der neue grüne Besen steht griffbereit da”). The correct answer for the experimental trials (congruent/incongruent) was always “Yes.” The correct answer for filler trials was always “No.” RA = Response accuracy; RL = Response latency.

In the main part of the experiment, 72 sentence–picture verification tasks were presented (without feedback). Each trial appeared in the same sequence (see Figure 1). A left-aligned fixation cross appeared on the screen for 1,000 ms, followed by the first word (black letters on a white screen). By pressing the space bar, participants moved to the next word. When all words had been displayed, a fixation cross appeared in the center of the screen for 500 ms. Then a picture was shown in the center of the screen. Participants responded with “j” for “true” or “f” for “false.” After the participants’ response, the picture disappeared, and then a blank screen was shown for 500 ms before the next trial began.

In half of the experimental trials (24 trials), the picture color was congruent to the color word (congruent condition). In the other 24 trials, the picture color was incongruent to the color word (incongruent condition). Each of the four color words (yellow, red, blue, green) occurred 12 times in the experimental trials. Each condition (congruent/incongruent) appeared six times per color, with the color word appearing three times in third place and three times in sixth place. All experimental trials (congruent and incongruent condition) were to be answered with “j” for “true.”

In the 24 filler trials, the object depicted was different from the object described. Each of the four color words occurred six times. All filler trials were to be answered with “f” for “false.”

For the experimental trials, two lists were created to ensure that each sentence occurred equally in combination with a congruent picture color and an incongruent picture color across participants. Within each list, the order of sentence presentation was randomized. In both lists, all fillers were additionally presented in a randomized manner. Participants were randomly assigned to one of the two lists.

After the experiment, the participants were presented with sentence verification tasks, focusing on microstructural reading processes (Effizienz des Leseverstehens bei Erwachsenen nach dem Strategiemodell [efficiency of reading comprehension in adult readers according to the strategy model]) (ELVES)﻿; Richter & van Holt, 2005), a mental comparison task that focused on mental imagery abilities (Suggate, 2024), and a questionnaire on mental imagery abilities (Plymouth Sensory Imagery questionnaire; Andrade et al., 2014). The data are included in the data files on OSF. These tasks were not relevant to the research questions of the current study. Thus, no results are reported for these tests. The entire experiment took about 30 min.

#### Design

The experiment was based on a one-factorial within-subject design with color match versus mismatch as the independent variable (congruent vs. incongruent colors of object picture and color word).

#### Data and Materials Availability

The full data, the R code for the model results as well as the experimental text material are available at the repository of the Open Science Framework (https://osf.io/7h2py/overview?view_only=3e46f51a4ee2437e826b8d2052e5fbb5). The full materials are available from the authors upon request.

### Results and Discussion

The reported analyses were conducted in R (Version 4.4.1.). All hypotheses were tested with a Type I error probability of α = .05. LMM were estimated for response latency as a dependent variable (Baayen et al., 2008) and generalized linear mixed models (GLMM) were estimated for response accuracy as a dependent variable (Jaeger, 2008). The models included the main effect of incongruent versus congruent condition (dummy coded 1 vs. 0). Random effects of participants and sentences were included (random intercepts). We used the R package lme4 to estimate the models (Bates et al., 2015). Follow-up analyses were conducted with the R package emmeans (Lenth, 2025).

**Figure 2.**
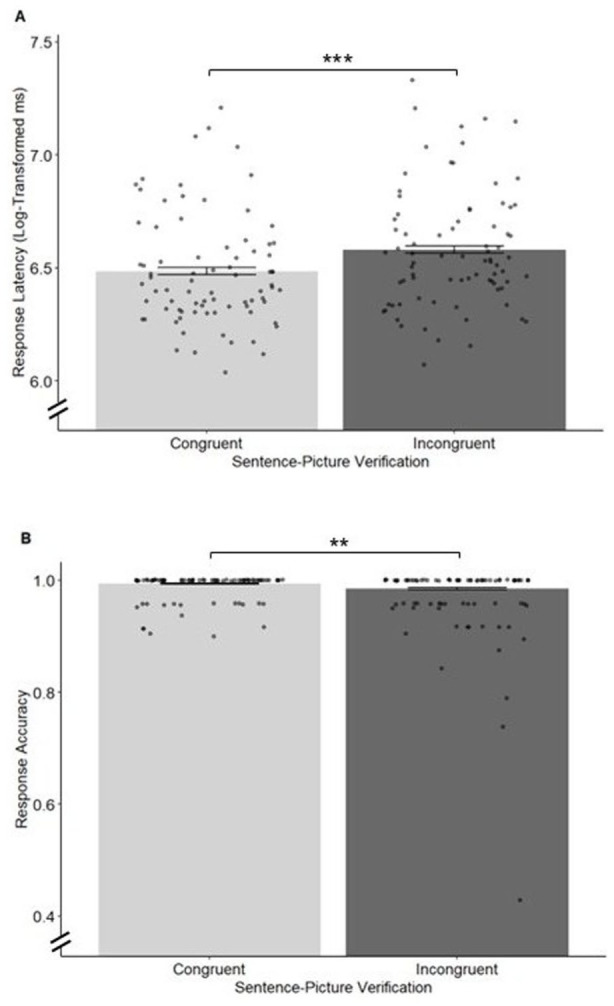
Mean response latency and response accuracy per condition in Experiment 1. *Note.* Mean response latency (only correct responses) (A) and mean response accuracy (B) for congruent and incongruent sentence–picture verification tasks. Error bars represent standard errors of the mean. ** *p* < .01. *** *p* < .001.

Results revealed the mismatch effect predicted by Hypotheses 1 and 2 (Figure 2). Responses in the incongruent condition were significantly slower (*M* = 6.580, Standard error [*SE*] = 0.032) than in the congruent condition (*M* = 6.484, *SE* = 0.032), β = 0.10, *SE* = 0.02, *t*(93) = 4.37, *p* < .001 (one-tailed), *d* = 0.37. Responses in the incongruent condition were also slightly but significantly less accurate (*M* = 0.985, *SE* = 0.004) compared to the congruent condition (*M* = 0.994, *SE* = 0.002), β = −0.92, *SE* = 0.32, *z* = −2.91, *p* = .002 (one-tailed), odds ratio (*OR*) = 0.40, 95% Confidence interval (*CI*) [0.21, 0.74].

Our results provide support for a routine, modality-based representation of color information in sentence comprehension when the color of the focal object is explicitly named in the sentence. The results correspond to the prototypical pattern of results in sentence–picture verification tasks by revealing a mismatch effect for color (consistent with Hoeben Mannaert et al., 2017; Zwaan & Pecher, 2012). However, this finding cannot be interpreted as evidence for a functional role of color representations during comprehension. Therefore, in Experiment 2, we provided competing color information during the reading process, which offers the opportunity of gaining causal insights into the functionality of perceptual simulations.

## Experiment 2

The aim of Experiment 2 was to investigate whether the mismatch effect for colors would be moderated by competing visually presented colors (background color) during the reading process (for the preregistration, see https://aspredicted.org/649x-s5qk.pdf). Based on neuroimaging findings suggesting that perceptual impressions and the comprehension of words that refer to the same perceptual dimension draw on the same cognitive resources (Barsalou, 1999; for color, see Simmons et al., 2007; Van Dam et al., 2012), visually presented conflicting colors should impair the comprehension of verbally described colors. Findings from behavioral interference paradigms also support this assumption. These include experiments with word–color interference tasks (for an overview, see Glaser, 1992). In this context, bidirectional congruence effects were found for visually presented colors and color words (Richter & Zwaan, 2009). Based on these findings we expected effects of conflicting color information on comprehension to be reflected in an altered response pattern in the sentence–picture verification task.

Specifically, we expected that when the picture color is congruent with the color word but incongruent with the background color (incongruent background color condition), participants would respond significantly slower (Hypothesis 1a) and less accurately (Hypothesis 1b) to a sentence–picture verification task than when all described and depicted colors were congruent (fully congruent condition). We also expected that when the picture color is congruent with the background color but incongruent with the color word (incongruent color word condition), participants would respond significantly slower (Hypothesis 2a) and less accurately (Hypothesis 2b) in a sentence–picture verification task than when all described and depicted colors are congruent (fully congruent condition). In the incongruent color word condition, the background and the picture colors are visually congruent, which is not the case in the incongruent background color condition. Therefore, the background color might prime the congruent picture color, facilitating “yes” responses in the sentence–picture verification task. Against this background, we assumed that responses in the incongruent background color condition, which included visual color incongruency, would be slower (Hypothesis 3a) and less accurate (Hypothesis 3b) than in the incongruent color word condition, which included color congruency.

### Method

#### Participants

We followed the power analysis for Experiment 1. Eighty-four datasets were collected. One dataset was excluded immediately after collection because the participant was taking part for the second time. Eleven participants were excluded because they answered less than 80% of the sentence–picture verification tasks correctly (according to our second exclusion criterion), which led to a final sample size of 72 participants (55 female, 15 male, 2 diverse), with an average age of 26.99 years (*SD* = 10.91). The majority of the participants were students (*n* = 64) of which 13 were psychology students, 23 were teacher students, and the remaining students (*n* = 28) were enrolled in other study programs.

#### Material

The same material as in Experiment 1 was used.

#### Procedure

**Figure 3.**
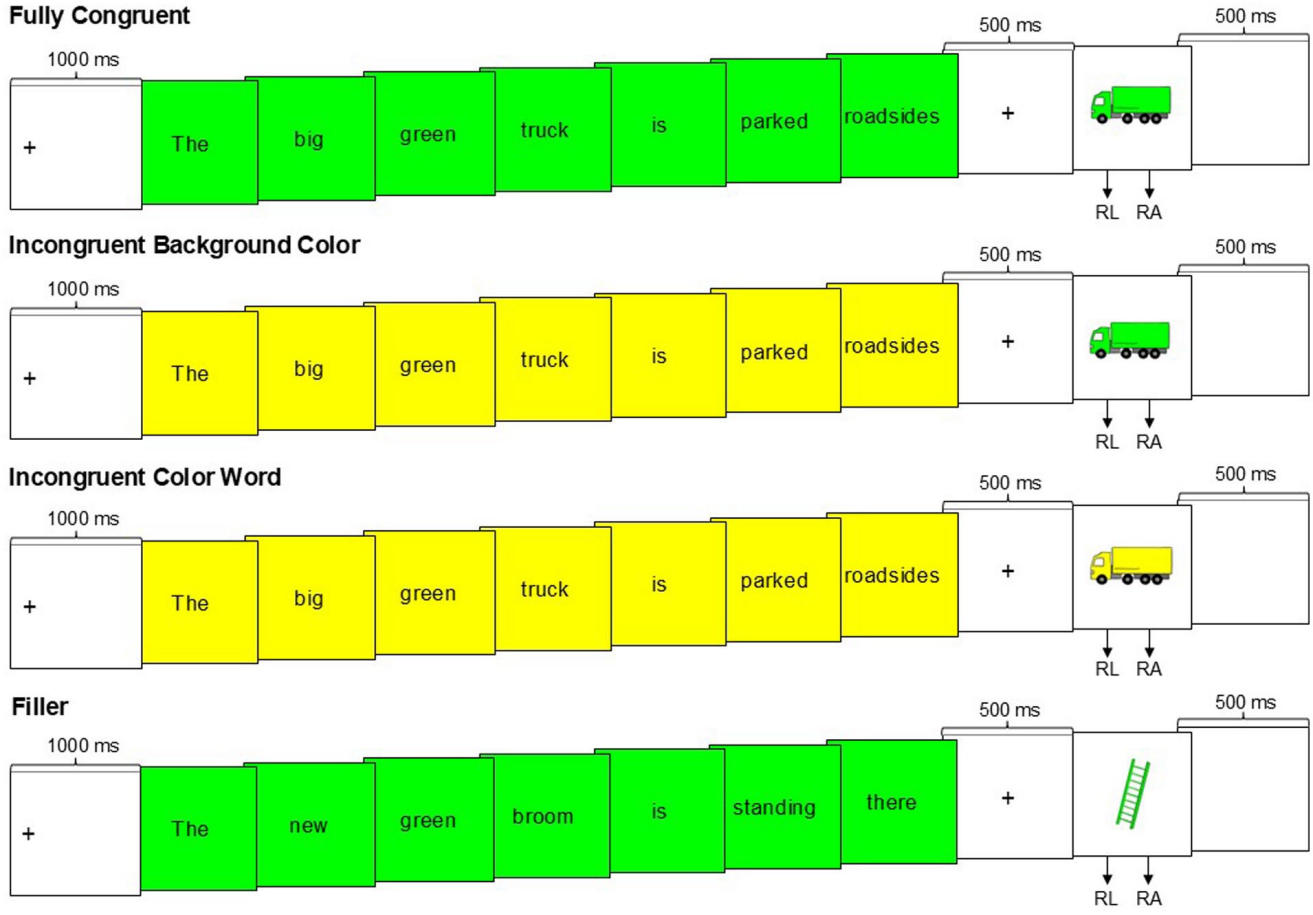
Example of the trial sequence in Experiments 2 and 3. *Note.* Each experimental sentence existed in three versions but only appeared once per list and per participant. The sentences were presented in German. The English translations of the sentences may not be exact, as they were intended to contain seven words like the German originals (original versions: “Der große grüne LKW steht am Straßenrand” and “Der neue grüne Besen steht griffbereit da”). The correct answer for all experimental trials (fully congruent/incongruent background color/incongruent color word) was always “Yes.” The correct answer for filler trials was always “No.” RA = Response accuracy; RL = Response latency.

The procedure was almost identical to the first experiment. The only difference was in the presentation of the sentences (see Figure 3). The experimental manipulation appeared in the form of background colors. Within a sentence, the same background color was presented for each of the seven words. The background colors represented pure instances of the RGB color model for yellow (255, 255, 0), red (255, 0, 0), green (0, 255, 0), and blue (0, 0, 255), analogous to the picture colors. The background colors were either congruent or incongruent with the color word and the picture color. This manipulation resulted in three conditions with 16 trials each:

- Fully congruent condition: The color word, the picture color, and the background color were congruent.
- Incongruent background color condition: The color word and the picture color were congruent, but the background color was an incongruent color.
- Incongruent color word condition: The picture color and the background color were congruent, but the color word was an incongruent color.

As in Experiment 1, 24 fillers were presented in which the object depicted differed from the object described. To keep the conditions parallel, we also displayed background colors for filler trials. In all filler trials, the background color matched the color word as well as the picture color. Again, all experimental trials were to be answered with “j” and all filler trials with “f.”

Each participant received 72 sentence–picture verification tasks. All sentences appeared once per participant. However, we ensured across all participants that each sentence appeared equally often in all three conditions. Therefore, we created three lists in which each sentence was assigned to another condition. Again, each of the four color words (yellow, red, blue, green) occurred 12 times per list. Each condition (congruent/incongruent background color/incongruent color word) appeared four times per color, with the color word appearing twice in third place and twice in sixth place. The 24 fillers from Experiment 1 were also presented and were identical for all participants. Participants were randomly assigned to one of the three lists. The sentences were presented in random order.

#### Design

The experiment was based on a one-factorial within-subject design with the independent variable color congruency (fully congruent condition vs. incongruent background color condition vs. incongruent color word condition).

### Results and Discussion

**Figure 4.**
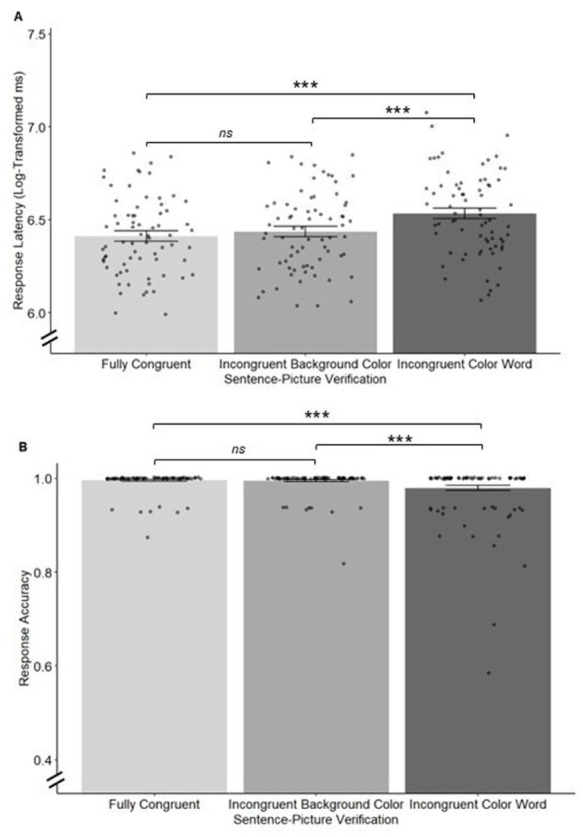
Mean response latency and response accuracy per condition in Experiment 2. *Note.* Mean response latency (only correct responses) (A) and mean response accuracy (B) for fully congruent, incongruent background color, and incongruent color word sentence–picture verification tasks. Error bars represent standard errors of the mean. *** *p* < .001.

We estimated an LMM for response latency and a GLMM for response accuracy (random intercept models with random effects of participants and sentences) with condition as a fixed factor (dummy-coded, with the fully congruent condition as the reference category coded 0). Pairwise comparisons between conditions were obtained using Holm-corrected contrasts (via the emmeans package). The means and standard errors for response latency and response accuracy by conditions are shown in Figure 4. Please note that this procedure (valid for Experiment 2 and 3) deviates slightly from the preregistrations, which describe the estimation of three separate (G)LMMs. The preregistered procedure yielded the same pattern of results, which are available in the Online Supplemental.

For response latency, the comparison of the incongruent background color condition (*M* = 6.436, *SE* = 0.028) with the fully congruent condition was not significant (*M* = 6.412, *SE* = 0.028), β = 0.02, *SE* = 0.02, *t*(138) = 1.15, *p* = .126 (one-tailed). For response accuracy, the comparison of the incongruent background color condition (*M* = 0.995, *SE* = 0.002) with the fully congruent condition (*M* = 0.996, *SE* = 0.002), β = −0.15, *SE* = 0.50, *z* = −0.30, *p* = .383 (one-tailed) was also not significant. Instead, participants made hardly any errors in both conditions. Thus, Hypotheses 1a and 1b received no support.

In a comparison of the incongruent color word condition and the fully congruent condition (Hypotheses 2a and 2b), responses in the incongruent color word condition were significantly slower (*M* = 6.534, *SE* = 0.028) than in the fully congruent condition (*M* = 6.412, *SE* = 0.028), β = 0.12, *SE* = 0.02, *t*(140) = 5.78, *p* < .001 (one-tailed), *d* = 0.51. Responses in the incongruent color word condition were also significantly less accurate (*M* = 0.981, *SE* = 0.006) compared to the fully congruent condition (*M* = 0.996, *SE* = 0.002), β = −1.58, *SE* = 0.41, *z* = −3.900, *p* < .001 (one-tailed), *OR* = 0.21, 95% CI [0.08, 0.54]. This pattern of results supports Hypotheses 2a and 2b.

In a comparison of the incongruent background color condition and the incongruent color word condition (Hypotheses 3a and 3b), responses in the incongruent background color condition were significantly faster (*M* = 6.436, *SE* = 0.028) than in the incongruent color word condition (*M* = 6.534, *SE* = 0.028), β = −0.10, *SE* = 0.02, *t*(139) = −4.63, *p* < .001 (two-tailed), *d* = 0.41. Responses in the incongruent background color condition were also significantly more accurate (*M* = 0.995, *SE* = 0.002) compared to the incongruent color word condition (*M* = 0.981, *SE* = 0.006), β = 1.44, *SE* = 0.39, *z* = 3.71, *p* < .001 (two-tailed), *OR* = 4.20, 95% CI [1.67, 10.61]. Both effects run counter to the effects predicted in Hypotheses 3a and 3b.

These unexpected results regarding Hypotheses 1 and 3 suggest that on the one hand, congruent background color and object color do not override the interference introduced by the incongruent color word in relation to the picture color. On the other hand, we found no evidence that an incongruent background color hampers responses in the sentence–picture verification task. This pattern of results suggests that the perceptual simulation of color information is less influenced by concurrently presented background information. Instead, color words that are incongruent with the picture color interfere with the sentence–picture verification task irrespective of background color. To examine the reliability of this finding, we conducted a direct replication study.

## Experiment 3

Experiment 3 was a direct replication of Experiment 2 to follow-up on the unexpected findings (preregistered at https://aspredicted.org/qdpk-n3kh.pdf). As for Experiment 2, we expected responses in the incongruent color word condition to be slower (Hypothesis 1a) and less accurate (Hypothesis 1b) compared to the fully congruent condition. The results of Experiment 2 furthermore suggested that an incongruent background color during sentence presentation does not affect responses in the sentence–picture verification task. We therefore expected responses in the incongruent color word condition to be slower (Hypothesis 2a) and less accurate (Hypothesis 2b) compared to the incongruent background color condition.

### Method

#### Participants

We followed the power analysis for Experiments 1 and 2. Eighty-four participants took part in the experiment. Fourteen participants were excluded because they answered less than 80% of the sentence–picture verification tasks correctly (according to our second exclusion criterion). Data from 70 participants were included in the analysis (age: *M* = 21.71 years, *SD* = 3.73; 58 female, 10 male, 2 diverse). All but one of the participants were students of which 20 were psychology students, 42 were teacher students, and the remaining students (*n* = 7) were enrolled in other study programs.

#### Material

The same materials were used as in Experiment 2.

#### Procedure

Experiment 3 followed the same procedure as Experiment 2.

#### Design

Experiment 3 was based on the same design as Experiment 2.

### Results and Discussion

**Figure 5.**
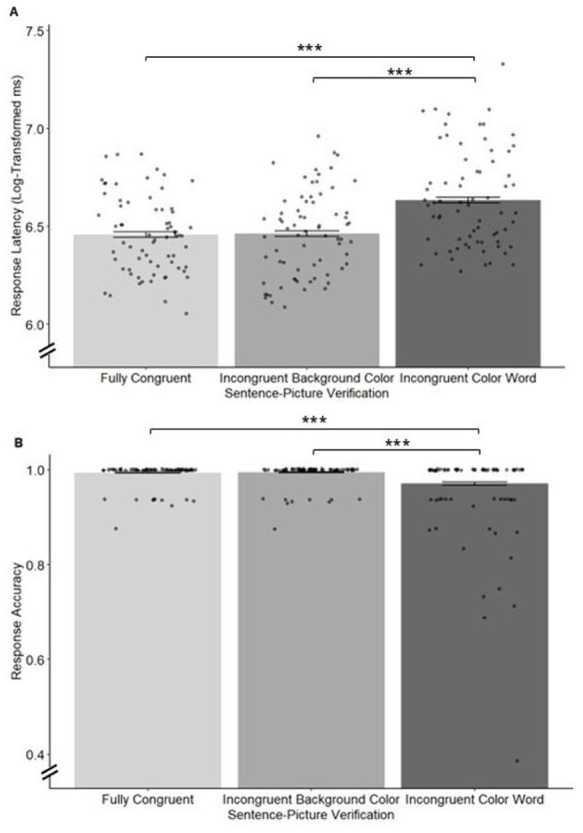
Mean response latency and response accuracy per condition in Experiment 3. *Note.* Mean response latency (only correct responses) (A) and mean response accuracy (B) for the fully congruent, incongruent background color, and incongruent color word sentence–picture verification tasks. Error bars represent standard errors of the mean. *** *p* < .001.

Again, we estimated an LMM for response latency and a GLMM for response accuracy (random intercept models with random effects of participants and sentences) with condition as a fixed factor (dummy-coded, with the fully congruent condition as the reference category coded with 0). Pairwise comparisons between conditions were obtained using Holm-corrected contrasts (via the emmeans package). The means and standard errors for response latency and response accuracy are shown in Figure 5.

The findings of Experiment 2 were replicated. Responses in the incongruent color word condition were significantly slower (*M* = 6.634, *SE* = 0.028) compared to the fully congruent condition (*M* = 6.459, *SE* = 0.028), β = 0.17, *SE* = 0.02, *t*(140) = 8.34, *p* < .001 (one-tailed), *d* = 0.67. Responses in the incongruent color word condition were also significantly less accurate (*M* = 0.971, *SE* = 0.008) compared to the fully congruent condition (*M* = 0.995, *SE* = 0.002), β = −1.70, *SE* = 0.36, *z* = −4.69, *p* < .001 (one-tailed), *OR* = 0.18, 95% CI [0.08, 0.41]. The pattern of results supports Hypotheses 1a and 1b.

For the comparison of incongruent color word condition and incongruent background color condition (Hypotheses 2a and 2b), the findings of Experiment 2 were also replicated. Responses in the incongruent color word condition were significantly slower (*M* = 6.634, *SE* = 0.028) compared to the incongruent background color condition (*M* = 6.463, *SE* = 0.028), β = 0.17, *SE* = 0.02, *t*(140) = 8.18, *p* < .001 (one-tailed), *d* = 0.66. Responses in the incongruent color word condition were also significantly less accurate (*M* = 0.971, *SE* = 0.008) compared to the incongruent background color condition (*M* = 0.995, *SE* = 0.002), β = −1.76, *SE* = 0.37, *z* = −4.70, *p* < .001 (one-tailed), *OR* = 0.17, 95% CI [0.07, 0.40]. The pattern of results supports Hypotheses 2a and 2b.

The mismatch effect remained regardless of whether the background colors were congruent with the color word or not. Responses in the incongruent color word condition were significantly slower and less accurate than in the incongruent background color condition. In line with Experiment 2, we found support for the assumption that color words that are incongruent with the picture color interfere with the sentence–picture verification task and the assumption that the perceptual simulation of color information is less influenced by concurrently presented visual color information.

## Exploratory Analyses of Reading Time Across Experiments 1 to 3

Since mental simulation is assumed to evolve during comprehension (Zwaan, 2004; Zwaan et al., 1998), we were interested in whether the mismatch effect would be affected by the presentation of incongruent colors, depending on how deeply participants engaged with the sentence during reading. To this end, we explored whether reading times within each condition differentially predicted response accuracy and/or latency in the sentence–picture verification task. In all experiments, the reading time per word was recorded during the self-paced presentation of the experimental sentences. We computed the sum scores per sentence (reading times per word over 4,000 ms were excluded beforehand as extreme outliers in each experiment) and included reading time as a second predictor (z-standardized) in the GLMMs and LMMs that were used to test these hypotheses. All conditions were included simultaneously for Experiments 2 and 3 (dummy-coded, with the fully congruent condition as the reference category coded with 0) and main effects and the interaction of the experimental conditions and the mean reading times were included simultaneously as fixed effects. We also included random effects (random intercepts) of participants and sentences.

**Figure 6.**
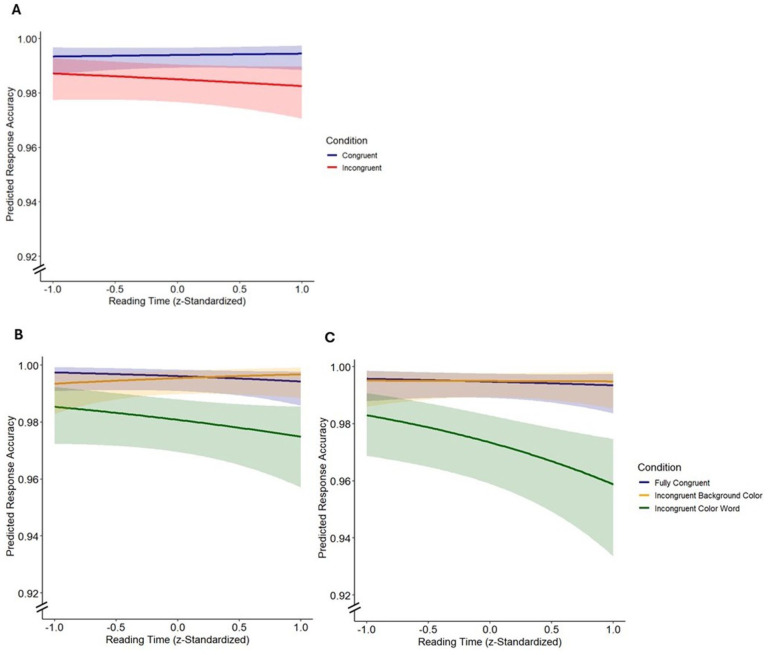
Estimated slopes of reading time predicting the probability of a correct response for Experiment 1 (A), Experiment 2 (B), and Experiment 3 (C) per condition. *Note.* Gray areas represent 95% CI per condition. CI = Confidence interval.

Based on these models, we conducted simple slope analyses to estimate condition-specific effects (Figure 6). The analyses revealed a significant negative slope in the incongruent color word condition in Experiment 3 for response accuracy (β = −0.45, *SE* = 0.17, *z* = −2.63, *p* = .009, two-tailed), suggesting that the longer participants read the sentences, the worse their response accuracy. Conversely, the less time that participants engaged with the sentences, the more they responded “yes” to picture colors that showed visual congruence with the background color, even though the picture color was incongruent to the color word, compared to participants who took more time to read the sentences. A similar trend emerged in the incongruent color word condition in Experiment 2, although it did not reach statistical significance (β = −0.28, *SE* = 0.18, *z* = −1.51, *p* = .132, two-tailed). In both experiments, no significant effects were observed for the fully congruent or incongruent background color condition (Table 1). In the incongruent condition in Experiment 1, which included no experimental manipulation of background color, reading times were not predictive of response accuracy (β = −0.16, *SE* = 0.16, *z* = −0.99, *p* = .320, two-tailed). This finding suggests that the negative effects of reading time on accuracy in the incongruent conditions of Experiment 2 and 3 were due to the processing of incongruent background colors in these experiments. Analogous analyses with response latency as the dependent variable showed positive slopes of reading time across all conditions (see Table A1 in the Online Supplemental), reflecting an overall close correlation of reading and reaction times.

**Table 1.** Simple Slopes of Reading Time on Response Accuracy per Condition Across All Experiments.

Condition per experiment	β	*SE*	*z*	*p*
Experiment 1				
Congruent	0.09	0.20	0.44	.663
Incongruent	–0.16	0.16	–0.99	.320
Experiment 2				
Fully congruent	–0.41	0.33	–1.23	.219
Incongruent background color	0.36	0.38	0.94	.350
Incongruent color word	–0.28	0.18	–1.51	.132
Experiment 3				
Fully congruent	–0.22	0.31	–0.70	.483
Incongruent background color	–0.03	0.34	–0.09	.932
Incongruent color word	–0.45	0.17	–2.63	.009

*Note.* All *p* values are based on two-sided tests. Reading time was z-standardized. SE = Standard error.

## General Discussion

In three preregistered experiments, we examined the role of perceptual simulations of color in the comprehension of sentences that contained color words. Our first objective was to examine whether a mismatch effect occurs when explicit color words are used in a sentence–picture verification task. Experiment 1 yielded the expected mismatch effect, extending previous results of sentence–picture verification tasks in the perceptual dimension of color (de Koning et al., 2017a; Hoeben Mannaert et al., 2017, 2021; Zwaan & Pecher, 2012). The results indicate that the color of focal objects is routinely activated during comprehension and also when color words are explicitly mentioned. Our experiments contribute to the growing body of literature supporting the assumption that color is activated in a similar manner to other perceptual dimensions during comprehension (see de Koning et al., 2017a) and not in a different manner as has been discussed in the past (see Connell, 2007). Methodologically, the use of color-matching fillers ensured that the response pattern was not based on strategic responses but instead were routine activations of sentence-related simulations, which is in line with previous research (van Zuijlen et al., 2024). Regarding embodied theories of language comprehension—particularly Barsalou’s (1999) theory of perceptual symbol systems—our results support the view that perceptual representations (specifically color representations) are routinely activated during comprehension.

Our second objective was to examine the functionality of perceptual simulations of colors in sentence comprehension. Together with the experimental sentences, we presented conflicting visual colors that we expected to impair the comprehension of color words, resulting in altered performance in the sentence–picture verification task. This expectation was not supported. Although color words that were incongruent with the picture color elicited significantly slower and less accurate responses than color words that were congruent with the picture color (corresponding to typical mismatch effect), this effect was not affected by the parallel processing of (conflicting) visual background color information. The results were supported in Experiment 3, a direct replication study with an independent sample.

We derive two main conclusions from the results. First, the mismatch effect found in Experiment 1 appeared to be robust across all experiments and was not affected by whether the visually presented colors were congruent with the color words or not. This finding indicates that color representations of focal objects are routinely activated during comprehension when their colors are explicitly mentioned, extending previous research on the continuous activation of color representations (Hoeben Mannaert et al., 2021). Second, the results suggest that the comprehension of color information is less influenced by concurrently presented visual color information than would have been expected based on the results of neuroimaging studies that have identified common processing areas for simulated and perceived color (Simmons et al., 2007; Van Dam et al., 2012) and studies that have demonstrated that the retrieval of visual information can be influenced by visual stimuli (Davis et al., 2020; Edmiston & Lupyan, 2017; Ostarek & Huettig, 2017). Based on these studies, we had assumed that the simulation of verbal color information can be influenced by visual color information when simulation is functionally involved in comprehension processes. However, this assumption was not supported by our results. Instead, our results are in line with findings by Ostarek et al. (2019) who manipulated the sentence–picture verification task by presenting visual shapes (“visual noise”) and irrelevant objects (“semantic noise”) during auditory sentence presentation. Visual noise did not affect the mismatch effect, whereas semantic noise did. The authors concluded that visual processes are not necessarily involved in the cognitive mechanisms underlying the mismatch effect and that this effect may therefore not be (primarily) grounded in modal simulations. In a similar approach, Buchner et al. (2025) examined the influence of direction-related perceptual stimuli on the processing of direction-related narrative stories and likewise found no match or mismatch effects. Consistent with these findings, we also found no evidence for a modulatory effect of visual processes—specifically, the processing of concurrently presented colors in this study—on the mismatch effect for colors. It may therefore be speculated that perceptual simulations are rather a “by-product” of comprehension (Strozyk et al., 2019, p. 407; for a theoretical discussion, see Goldinger et al., 2016).

An alternative interpretation of the unexpected results is offered by theories of conflict processing. According to the conflict monitoring theory proposed by Botvinick et al. (2001), information processing is continuously monitored to detect potential conflicts between competing response options (e.g. as in Stroop tasks). When such a conflict is detected, cognitive control is upregulated, leading to heightened attention and more accurate responses. In line with this framework, and contrary to our hypotheses in Experiment 2, that parallel processing of conflicting background colors would reduce response accuracy and increase response latency, the simultaneous visual presentation of conflicting colors may instead have enhanced attention and comprehension, thereby accounting for the absence of an interference effect in Experiment 2.

Although no measurable direct effects of visual background color on response latency and accuracy were found, exploratory analyses revealed differences between the conditions when considering reading time as an indirect index of processing effort during sentence reading. In the incongruent color word condition, when color word and picture color were incongruent, but picture color was concurrently congruent with the background color, the mismatch interference was reduced (i.e. response accuracy was less deteriorated) for participants who read faster compared to participants who read more slowly. Assuming that shorter reading times indicate more superficial reading processes and longer reading times indicate deeper reading processes, participants who processed the sentences more deeply might have been more likely to be affected by the incongruency between color word and picture color and less likely to be affected by the background color because they were engaged in a thorough simulation of the sentence content, resulting in a comparatively stronger mismatch interference. The exploratory findings therefore suggest that superficial reading processes are more strongly influenced by concurrently presented colors than deep reading processes, whereas deep reading processes might be accompanied by more resistant simulations. In addition, the conflict monitoring theory proposed by Botvinick et al. (2001) can again be applied to explain that participants who read more slowly may have perceived the color conflict, thereby engaging higher cognitive control, in contrast to those who spent less time on the sentence.

As the incongruent color word condition is the only one involving a word–picture mismatch, an important alternative interpretation is that the reading time effect might reflect the perceived semantic incongruence instead of the interference of background color. It seems reasonable that longer reading times (or deeper processing) are associated with increasing salience of semantic incongruence. At this point, the lack of a fully crossed design prevents a clear interpretation of this effect.

However, the effect did not occur in Experiment 1, which also required responses to incongruent picture colors but with no experimental manipulation in the form of background colors, making this explanation unlikely. In sum, the exploratory findings on reading time support the interpretation that incongruent background colors can affect perceptual simulations in sentence comprehension but only when readers process the sentence superficially. Given that this interpretation is based on exploratory findings, it remains to be tested in confirmatory studies.

### Limitations and Future Directions

One limitation of our findings is that our experimental designs were reduced to keep the procedure short and avoid habituation or practice effects. The downside of the reduced design is that our method lacked a fully crossed design of congruent/incongruent background color and congruent/incongruent object color. Such a design might have enabled more comprehensive conclusions. A second limitation is that despite this precaution, we cannot rule out the possibility that participants became habituated to the repeated presentation of visual colors. Even in simple Stroop tasks, habituation effects have been observed following frequent task repetition (see Stroop, 1935, Experiment 3). Therefore, habituation effects might have prevented the effects of background color from occurring. A third limitation is that the reading-time-dependent effect of congruent versus incongruent background color on the mismatch effect was observed only for response accuracy, not response latency, and was based on exploratory analyses, requiring confirmatory testing in future studies.

Future research should therefore further investigate the role of visual interference during on-line reading, depending on the depth of sentence processing, as this focus remains a promising yet underexplored avenue for understanding the mental processes involved in reading. Further studies are required to investigate the role of simulations in various perceptual areas and to draw more far-reaching conclusions about their significance in language comprehension.

## Conclusion

Our results indicate that the mismatch effect for sentences containing color words remains robust even when an incongruent background color is presented concurrently during reading. This finding suggests that the comprehension of explicit verbal color information is less susceptible to interference from simultaneously presented visual color information than might be expected based on findings from neuroimaging studies. Overall, the results of the present experiments provide evidence that color representations are routinely activated during language comprehension when colors are explicitly mentioned.

## Supplemental Material

sj-pdf-1-qjp-10.1177_17470218261428130 – Supplemental material for The Processing of Color Words in Sentence Comprehension Supplemental material, sj-pdf-1-qjp-10.1177_17470218261428130 for The Processing of Color Words in Sentence Comprehension by Emily L. Buchner, Tobias Richter and Wolfgang Lenhard in Quarterly Journal of Experimental Psychology
