# Supplementary material for: The Processing of Color Words in Sentence Comprehension
Source: Q J Exp Psychol (Hove). 2026 Feb 20;79(8):2242–58. doi: 10.1177/17470218261428130 (PMC13400818; doi:10.1177/17470218261428130)
Supplement: sj-pdf-1-qjp-10.1177_17470218261428130 – Supplemental material for The Processing of Color Words in Sentence Comprehension [file sj-pdf-1-qjp-10.1177_17470218261428130.pdf]

Experiment 2

The following section presents the results of the analyses that were preregistered for Experiment 2 (for the preregistration see https://aspredicted.org/649x-s5qk.pdf). Please note that the procedure reported in the manuscript for Experiment 2 deviates slightly from the preregistrations but yields the same pattern of results.

Results and Discussion

We estimated LMMs for response latency and GLMMs for response accuracy (random intercept models with random effects of participants and sentences).

To compare the incongruent background color condition and the fully congruent condition (Hypotheses 1a and 1b), we included the effect of incongruent background color condition vs. fully congruent condition (dummy-coded 1 vs. 0) in the LMM and GLMM.

For response latency, the comparison of the incongruent background color condition ($M = 6.433$, $SE = 0.010$) with the fully congruent condition was not significant ($M = 6.411$, $SE = 0.010$), $\beta = 0.02$, $SE = 0.02$, $t(2207) = 1.08$, $p = .142$ (one-tailed). For response accuracy, the comparison of the incongruent background color condition ($M = .992$, $SE = 0.003$) with the fully congruent condition ($M = .993$, $SE = 0.003$), $\beta = -0.10$, $SE = 0.57$, $z = -0.17$, $p = .433$ (one-tailed) was also not significant. Instead, participants made hardly any errors in both conditions. Thus, Hypotheses 1a and 1b received no support.

To compare the incongruent color word condition and fully congruent condition (Hypotheses 2a and 2b), we estimated a separate LMM and GLMM with the effect of incongruent color word condition vs. fully congruent condition (dummy-coded 1 vs. 0).

Responses in the incongruent color word condition were significantly slower ($M = 6.506$, $SE = 0.010$) compared to the fully congruent condition ($M = 6.411$, $SE = 0.010$), $\beta = 0.11$, $SE = 0.02$, $t(2144) = 4.93$, $p < .001$ (one-tailed), $d = 0.52$. Responses in the incongruent color word condition were also significantly less accurate ($M = .968$, $SE = 0.005$) compared to the fully congruent condition ($M = .993$, $SE = 0.003$), $\beta = -1.61$, $SE = 0.40$, $z = -4.01$, $p < .001$

(one-tailed), *OR* = 0.20, 95% CI [0.09, 0.44]. This pattern of results supports Hypotheses 2a and 2b.

To compare the incongruent background color condition and incongruent color word condition (Hypotheses 3a and 3b), we estimated a separate LMM and GLMM with the effect of incongruent background color condition vs. incongruent color word condition (dummy-coded 1 vs. 0).

Responses in the incongruent background color condition were significantly faster (M = 6.433, SE = 0.010) compared to the incongruent color word condition (M = 6.506, SE = 0.010), β = -0.08, SE = 0.02, $t(2144) = -4.07$, $p < .001$ (two-tailed), d = 0.42. Responses in the incongruent background color condition were also significantly more accurate (M = .992, SE = 0.003) compared to the incongruent color word condition (M = .968, SE = 0.005), β = 1.44, SE = 0.39, z = 3.68, $p < .001$ (two-tailed), *OR* = 4.22, 95% CI [1.96, 9.10]. Both effects run counter to the effects predicted in Hypotheses 3a and 3b.

Experiment 3

The following section presents the results of the analyses that were preregistered for Experiment 3 (preregistered at https://aspredicted.org/qdpk-n3kh.pdf). Please note that the procedure reported in the manuscript for Experiment 3 deviates slightly from the preregistrations but yields the same pattern of results.

Results and Discussion

Again, we estimated LMMs for response latency and GLMMs for response accuracy (random intercept models with random effects of participants and sentences).

To compare the incongruent color word condition and fully congruent condition (Hypotheses 1a and 1b), we included the effect of incongruent color word condition vs. fully congruent condition (dummy-coded 1 vs. 0) in the LMM and GLMM.

The findings of Experiment 2 were replicated. Responses in the incongruent color word condition were significantly slower (*M* = 6.600, *SE* = 0.011) compared to the fully congruent condition (*M* = 6.458, *SE* = 0.010), β = 0.15, *SE* = 0.02, $t(2065) = 7.61$, $p < .001$ (one-tailed), *d* = 0.80. Responses in the incongruent color word condition were also significantly less accurate (*M* = .953, *SE* = 0.007) compared to the fully congruent condition (*M* = .990, *SE* = 0.003), β = -1.69, *SE* = 0.35, *z* = -4.87, $p < .001$ (one-tailed), *OR* = 0.18, 95% CI [0.09, 0.36]. The pattern of results supports Hypotheses 1a and 1b.

For the comparison of incongruent color word condition and incongruent background color condition (Hypotheses 2a and 2b), we estimated a separate LMM and GLMM with the effect of incongruent color word condition vs. incongruent background color condition (dummy-coded 1 vs. 0).

The findings of Experiment 2 were also replicated for this comparison. Responses in the incongruent color word condition were significantly slower (*M* = 6.600, *SE* = 0.011) compared to the incongruent background color condition (*M* = 6.460, *SE* = 0.011), β = 0.15, *SE* = 0.02, $t(2065) = 6.85$, $p < .001$ (one-tailed), *d* = 0.72. Responses in the incongruent color word condition were also significantly less accurate (*M* = .953, *SE* = 0.007) compared to the incongruent background color condition (*M* = .991, *SE* = 0.003), β = -1.75, *SE* = 0.37, *z* = -4.72, $p < .001$ (one-tailed), *OR* = 0.17, 95% CI [0.08, 0.36]. The pattern of results supports Hypotheses 2a and 2b.

Table A1

Simple Slopes of Reading Time on Response Latency per Condition Across All Experiments

Condition per Experiment	β	*SE*	df	*t*	*p*
Experiment 1					
Congruent	0.08	0.01	3529	10.07	<.001
Incongruent	0.06	0.01	3532	7.85	<.001
Experiment 2					
Fully Congruent	0.08	0.01	3145	8.03	<.001
Incongruent Background Color	0.05	0.01	3142	5.70	<.001
Incongruent Color Word	0.04	0.01	3149	3.92	.001
Experiment 3					
Fully Congruent	0.12	0.01	3009	11.32	<.001
Incongruent Background Color	0.11	0.01	3021	11.01	<.001
Incongruent Color Word	0.12	0.01	3025	11.48	<.001

Note. All *p*-values are based on two-sided tests. Reading time was z-standardized.
